# The justification for strike action in healthcare: A systematic critical interpretive synthesis

**DOI:** 10.1177/09697330211022411

**Published:** 2022-04-12

**Authors:** Ryan Essex, Sharon Marie Weldon

**Affiliations:** University of Greenwich, UK

**Keywords:** Healthcare, nurse, protest, strike, strike action

## Abstract

Strike action in healthcare has been a common global phenomenon. As such action is designed to be disruptive, it creates substantial ethical tension, the most cited of which relates to patient harm, that is, a strike may not only disrupt an employer, but it could also have serious implications for the delivery of care. This article systematically reviewed the literature on strike action in healthcare with the aim of providing an overview of the major justifications for strike action, identifying relative strengths and shortcomings of this literature and providing direction for future discussions, and theoretical and empirical research. Three major themes emerged related to (1) the relationship between healthcare workers, patients and society; (2) the consequences of strike action; and (3) the conduct of strike action. Those who argue against strike action generally cite the harms of such action, particularly as it relates to patients. Many also argue that healthcare workers, because of their skills and position in society, have a special obligation to their patients and society more generally. Those who see this action as not only permissible but also, in some cases, necessary have advanced several points in response, arguing that healthcare workers do not necessarily have any special obligation to their patients or society, and even if so, this obligation is not absolute. Overwhelmingly, when talking about the potential risks of strike action, authors have focused on patient welfare and the impact that a strike could have. Several directions for future work are identified, including greater explorations into how structural and systemic issues impact strike action, the need for greater consideration about the contextual factors that influence the risks and characteristics of strike action and finally the need to tie this literature to existing empirical evidence.

## Introduction

A strike is a collective action, that generally involves a temporary stoppage of work to raise a grievance or as a means to have some kind of demand met.^1^ Over the last century, strike action has been a common occurrence, throughout the world and among healthcare professions. As strikes are calculated to disrupt, they raise a range of distinct dilemmas when undertaken by healthcare workers. That is, a stoppage of work by healthcare workers, unlike a number of other professions, may not only disrupt an employer, but such action could also have serious consequences for patient care.

While the impact that a strike may have on patients is often the first issue that comes to mind, a range of further issues present themselves. How a strike is conducted, the demands made, the risks to strikers themselves and even how such action is received by the public, all play into a series of practical and ethical considerations regarding the justifiability of such action. We can find examples of each of these concerns in healthcare strikes, with strikes varying substantially. The length of strikes carried out by healthcare workers has lasted anywhere from a number of hours, up to hundreds of days, as was the case with the 2016–2017 doctor and nurse strikes in Kenya.^2^ While the demands made generally relate to some type of workplace dispute, often to pay and conditions or patient care, strikes have been conducted for a range of other reasons. For example, in India, doctors went on strike for 3 weeks in 2006 because of government plans to boost the numbers of people from ‘low castes’ that were admitted to state-funded colleges.^3^ While strikes generally end peacefully, this is not always the case; in Pakistan, in response to a strike by junior doctors in 2012, the police raided several hospitals in an attempt to break up the strike, ‘arresting, attacking, and humiliating’^4^ hundreds in the process. In addition to varying substantially, strike action is almost always dynamic, with demands and risks shifting as a strike progresses. Many of these factors are further influenced by the circumstances and context in which they occur. Some strikes have been carried out with contingencies for patient care in place, while others have not.^5^ Strikes have also occurred in a range of healthcare systems, all resourced and structured differently. In addition to all of these things, there remains the epistemic uncertainty that strike action entails, that is, we can never be quite sure about how a strike will play out or the harm it may cause.

Perhaps unsurprisingly then, strike action has long been debated in the bioethics literature. Discussions have often been passionate and polarised, often flaring around episodes of strike action. Despite this, there remains little consensus on whether strike action is justified and if so, how we justify such action. This article sets out to systematically search the literature on strike action in healthcare with the overarching aim of providing an overview of the major justifications for strike action in healthcare, identifying relative strengths and shortcomings of this literature and providing direction for future discussions, and theoretical and empirical research. We hope that this will provide a foundation for discussion on decision-making in relation to healthcare strike action.

## Methods

### Design

This article employed a systematic search and critical interpretive synthesis. This type of review draws on techniques from more traditional systematic reviews and grounded theory.^6^ Unlike more traditional systematic reviews and forms of synthesis, an interpretive synthesis is concerned with the development of concepts and theory, utilising both induction and interpretation in the synthesis of data.^7^ A critical interpretive synthesis is particularly well suited to the field of bioethics and was well suited to our research question. While our search was systematic, we have not attempted to include and synthesise every article that deals with the justifiability of strike action. Unlike the broader healthcare literature, which may be concerned with the effectiveness of an intervention and thus where it is necessary to assemble all available evidence, research questions in bioethics differ. That is, questions predominantly focus on the justification of an action or in understanding the most salient normative elements of an issue, for example. Thus, research questions in bioethics do not rely on data in the same way as other studies, while additional evidence may affect studies focused on effectiveness; they may add little argument about ethical justifiability.^8^

Consistent with this approach, this review took the following steps: (1) framing the research question, (2) literature selection, (3) quality appraisal, (4) data extraction and (5) data synthesis. Each of these steps is expanded upon below.

### Research questions

What are the reasons given in the literature regarding the justifiability of strike action in healthcare? What are the relative strengths and shortcomings of this literature and what direction does this provide for future discussions, and theoretical and empirical research?

### Search strategy

While a critical interpretive synthesis generally allows a degree of flexibility in relation to a search, allowing a search strategy to emerge organically,^7^ after a number of preliminary searches, we found that a structured search served the needs of the research questions providing a comprehensive sample of papers. Search terms were developed to capture the core concepts, related to the form of action we were interested in (e.g. strike action, industrial action) and the populations in question (e.g. doctors, nurses, healthcare workers). While not exhaustive, preliminary searches that explored these terms suggested that they gave us substantial coverage of the literature in which we were interested. The final search terms were: strike OR ‘industrial action’ OR ‘industrial dispute’ OR ‘collective action’ AND doctor OR physician OR clinician OR ‘medical practitioner’ OR nurs* OR ‘health profession*’ OR healthcare OR ‘health care’ OR ‘pharmac*’ OR ‘dentist’ OR ‘midwi*’ OR ‘health worker’ OR ‘hospital’. A search was undertaken on 19 November 2020 using Scopus, Web of Science, CINAHL, Medline and PsycInfo. The reference lists of included papers were also searched for relevant articles.

### Search results and literature selection

**Figure 1.**
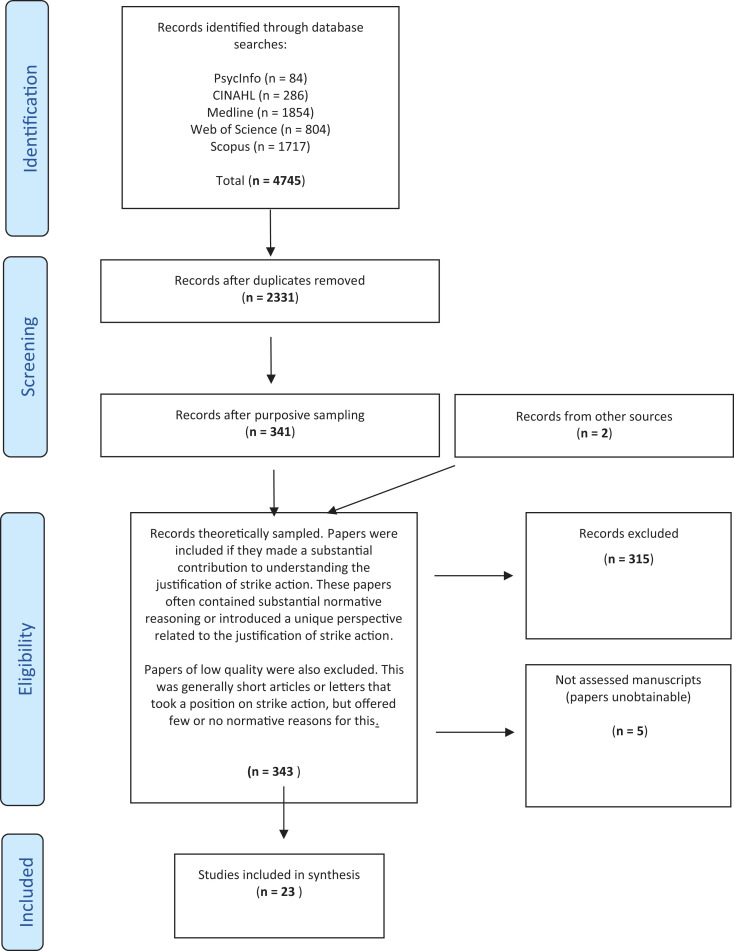
Modified PRISMA flow diagram.^9^

The above search yielded 4745 results. There were 2331 article after duplicates were removed. Unlike more traditional systematic reviews, in examining which papers to include/exclude, we did not apply rigid inclusion/exclusion criteria to these results, instead we employed an approach outlined by Dixon-Woods et al.^7^ In this case, a more rigid inclusion/exclusion criteria would have been inappropriate as the boundaries of this literature were relatively diffuse. Therefore, to limit the papers in this search, we first applied purposive sampling to select papers that were most relevant to our research question, generally scanning titles and abstracts of articles. This left us with 341 papers, to which we theoretically sampled. Generally, papers were included if they made a substantial contribution to understanding the justification of strike action. These papers often contained substantial normative reasoning or introduced a unique perspective related to the justification of strike action. This continued concurrently with theory generation.^7^ This left us with 23 papers. Many of the articles excluded at this point were letters to the editor, correspondence or short opinion pieces, many of which took a stance on strike action (and often putting forth a clear position for or against strike action), however offered little new on the reasons for why strike action may or may not be justified. Articles were also excluded if serious deficits were identified (see below). See Figure 1 below.

### Quality appraisal

While more traditional systematic reviews conduct a quality appraisal for each of the included papers, such an approach presents difficulties in bioethics.^8^ In short, the criteria on which this literature could be judged are substantially different to those of empirical studies. For this reason, we have again employed a similar approach to Dixon-Woods et al.,^7^ that is, while theoretically sampling papers, we were also mindful of their contribution to the literature and the arguments they offered. Papers that had significant flaws were initially excluded. In this case, the majority of papers that were automatically excluded were short articles or letters that took a position on strike action, but offered little or no normative reasons for this. Many that fell into this category simply asserted that strike action could not be justified, as it would impact negatively on patient well-being, with little consideration given to other dimensions of this problem. For the remainder of the papers, we were mindful about their credibility and contribution to our research questions. Instead of using the quality of these articles as a precursor to their inclusion, we have critiqued both individual papers and this literature as a whole in our results and discussion sections.

### Data extraction and synthesis

A data-extraction pro-forma was devised that identified the study, a summary of its major arguments and the major themes that emerged from the paper. This pro-forma was constantly amended to accommodate for emerging themes and to consolidate sub-themes into overarching themes. Data were synthesised with the aim of creating a ‘synthesising argument’. That is, the integration of evidence into ‘a coherent theoretical framework comprising a network of constructs and the relationships between them’.^7^ Themes that were most powerful in representing the data were identified through constant comparison. We then developed an argument that integrated the evidence from across the literature. In our case, this was done with our above research questions in mind. The articles that were included in this review, along with their major arguments, ideas and themes are summarised in Table 1.

**Table 1.** Summary of articles included in this review and their major arguments/ideas/themes.

Authors	Year	Summary	The relationship between healthcare workers, patients and society	The consequences of strike action	Conducting strike action
Brecher^10^	1985	This article argues that healthcare workers are not under any special obligation to refrain from going on strike, taking on a major argument that healthcare strike are unique as healthcare workers have a special responsibility to their patients. The author argues that strikes are not necessarily a good thing or the best means to solve dilemmas; however, as healthcare workers have no ‘special responsibility’ to their patients, they are a permissible form of action. More so, the authors argue that it is in fact those arguing against strike action ‘those who bear the greatest responsibility, on their own grounds, for needless death and suffering’	This article centres on the question of whether strike action can be justified. The author argues that ‘workers are not under any special obligation to refrain from going on strike’, on the ‘grounds that their circumstances as medical workers are not relevantly special’. The author goes on to argue that unless ‘human life is in all circumstances a completely overriding value…the striker whose omissions bring about someone’s death has no prima facie moral case to answer’		
Chima^11^	2013	This article discusses a range of issues related to strike action. Interestingly this article introduces a number of issues that are particularly pertinent to health in Africa and ties the issues of strikes in with issues such as brain drain. The author argues strongly for strike action, however acknowledges that health workers should consider patient safety and put safeguards in place if taking strike action		While the author suggests that healthcare workers should consider the impact of a strike on patients, the author also believes that the government also has responsibility, arguing that they hold the same responsibility for healthcare	This article also discusses a number of characteristics of strike action, such as the aims of strike action, arguing that ‘doctors and other workers must resist the impulse to make economic demands which are beyond the capacity of the employer or which could hamper the provision of other social services’. The article also calls on healthcare workers to provide a minimum standard of care if they go on strike
Counihan^12^	1982	While sympathetic to strike action, this author argues strongly against it, citing the potential impact it may have on patients as a primary concern. The author instead calls for a number of reforms aimed at avoiding strike action	This author argues that there is no basis for strike action, mainly because of the potential it has to harm patients	While the author acknowledges that ‘[t]here are obviously gradations in the consequence of withdrawal of service’ they argue against a strike on the grounds that it could harm patients, noting that ‘if management is doing its job properly, there are no non-essential workers in the Health Service’	This article dismisses the idea that providing care during a strike is possible, arguing that this ‘is a very nebulous concept’
Daniels^13^	1978	This article discusses the issue of collective bargaining, unionisation, professionalism and strikes. In relation to the justification of strikes, this article focuses on the reasons for striking (under the assumption that physicians are generally well paid) and discusses a number of characteristics of strike action. The author suggests that strike action can be justified if there are no serious risks to patients		This article argues a strike can be justified if it presents no serious risks to patients. Unlike a number of other papers here, the author discusses the potential conflict between unionisation and professionalism	This article argues that the demands of a strike are far more important than arguments related to the justification for such action. The author notes that they would find it hard to justify a strike if it ‘did not have as a significant part of their goals demands directly related to improved patient care’. The author also discusses some other issues, like strike action being a last resort and considering the degree of public support that the strike receives
Dimond^14^	1997	This article reviews the regulatory and legal issues related to a strike for nurses in the United Kingdom. This article discusses how nurses may be held accountable if taking strike action		This article explores the law relating to strikes and other industrial action in the United Kingdom and the problems faced by nurse practitioners. It also reviews the advice given to nurses by the professional associations. If any employee takes part in industrial action, he or she could personally face four arenas of accountability for this action: disciplinary proceedings before the employer, criminal proceedings, civil proceedings for negligence and professional conduct proceedings	
Dworkin^15^	1977	This article examines the moral and legal arguments related to strikes within the medical profession. The author argues that there is no justification for strike action and largely focus on two points, the harm to patients and a broader harm to society that a strike may promote		This article argues that ‘grief, distress, physical harm and, almost certainly, unnecessary death’ almost always occur as a result of strike action. The author goes on to dismiss arguments for strike action that maintain that emergency is left in place. Interestingly and unlike many other articles, here the author argues that a strike could prompt broader harm through promoting disobedience towards the law and ‘upset dramatically the social and political balance of the country’	
Fiester^16^	2004	This article offers three related arguments to support a prima facie prohibition against strike action. The author argues that strikes are intended to cause harm to patients; strikes are an affront to the physician–patient relationship and strikes risk decreasing the public’s respect for the medical profession. The author argues that a strike could be justified in very limited circumstances	This article opposes strike action on number of grounds; interestingly and in contrast to some of the work above, the author argues that strike action is an ‘affront to the physician-patient relationship’	This article opposes strike action in relation to the risks they present. The author not only argues that strike action has the potential to harm patients, but that strike action intentionally harms patients. The author also argues that strike action also has the potential to damage the doctor–patient relationship more generally and the general public’s respect for the medical profession	Interestingly and unlike many other articles, here this article argues that a strike could be justified (or more justifiable) if patient consent was obtained. The article argues that ‘[r]ather than this strike being a case of promise-breaking, it is a case of patients’ temporarily releasing physicians from a contractual agreement’
Glick^17^	1986	This article was written in response to Brecher (above), and essentially takes on a number of Brecher’s points arguing that a strike is never justified ‘regardless of the provocation’	This article argues that healthcare workers are in a ‘special class’ because they deal with human lives and because, upon joining the profession or accepting their job, they have voluntarily undertaken a commitment to those they serve	This article argues that strike action cannot be justified, mainly because of the risks it presents to patients; the author offers the analogy that strike action from healthcare workers is like ‘airline pilots threaten[ing] to parachute from their planes and leave their passengers without a pilot in mid-air’	
Jackson^18^	2000	This article explores medical strikes in relation to trust. That is, how a strike impacts on trust of medical professionals and the medical profession more broadly. The author argues that the complex nature of the trust relationship between physicians and patients is, in large part, why healthcare strikes are so problematic. The author suggests that strikes could be justified pending how they are conducted, but gives little detail on how to ‘conduct’ a justified strike		Rather than focus on risks to health, this article focuses on how a strike may be perceived and the role this may have in its justification. This article argues that strike action could have long-term impacts on how the public perceive the professions. The author argues that this could cut both ways, noting that if done for the ‘right’ reasons, strike action may preserve professional identities ‘as healers’. Equally, however, a strike could lead to patients feeling betrayed by healthcare workers	
Johnstone^19^	2012	This brief article introduces a unique perspective in that it shows how the idea of ‘patient safety’ can be co-opted. The author shows how, during strikes in Australia, the government manipulated concerns about patient safety to ‘name, blame and shame’ nurses		This article provides an example of strike action in Australia and raises a number of interesting questions about the responsibility for such action, along with how this was manipulated by the Australian government. The author notes that ‘the government of the day repeatedly used “patient safety” to name, blame and shame the nurses for their action and to falsely attribute the “everyday” deficits and failings of the healthcare system to the industrial action being taken’. This article shows how arguments about patient care can be made to support and oppose strike action	
Li et al.^20^	2015	This article considers a range of factors that justify strike action. The authors argue that for strikes to be considered justified, a minimum standard of care for patients should remain in place, the action should aim to improve care for future patients and that no alternatives exist to address the issues at hand		In relation to the risks of strike action, the authors introduce a temporal aspect and again show the malleability of the idea of using ‘patient care’ as a means to argue for and against strike action. The authors argue that, at times, ‘advocating for “best care” for future patients may mean compromising on “best care” for current patients’. They go on to argue that there are already precedents for this, for example, replacing facilities may reduce capacity in the shorter term but lead to better care in the longer term	This article assumes that strike action should be undertaken to improve patient care over the longer term, it does not discuss whether other demands could be justified, however does acknowledge that strikes often have multiple and mixed goals. The authors also argue that a strike should leave in place a minimal standard of care and that for this reason it would be difficult to justify a complete withdrawal of all staff. They also argue that a strike should only occur after all alternatives have been exhausted if it is to be justified
Loewy^21^	2000	This article presents a somewhat unique perspective, arguing that healthcare is not the most important social good and that healthcare professionals are not any more essential than a range of other workers (somewhat similar to Brecher above). The author argues that while some of the services provided by healthcare workers are life-saving, many are not. The authors argue that four particular elements of strikes should be singled out for scrutiny: the nature of the work, the prior commitment of the striking worker to the person served or to be served, the particular situation extant when such a strike is contemplated and the person or persons whom such a strike is meant to benefit	One focus of this article relates to the permissibility of strike action. The author argues that to maintain a strike is not justifiable one also has to maintain that ‘healthcare is a paramount human value’. The author argues that this could result in healthcare workers having to continue to work under any circumstance. Unlike Brecher above, this article does not maintain that healthcare workers have no special obligations, the article does acknowledge that healthcare workers play important roles, but that the obligations attached to these roles have limits	The author does discuss the issue of the risks that strike action presents, noting that ‘under most circumstances, are not free simply to “walk out” and abandon critically ill patients to their own devices…Only as a last resort, and that under almost inconceivable conditions, might a total strike of healthcare workers be justified’	This article discusses the demands attached to strike action. Unlike a number of other articles, the author argues that strike can be justified if it is carried out in self-interest, that is, better pay or working conditions. The author also indirectly addresses the question of who should go on strike, noting that a total strike (involving all professionals) could only be justified as a last resort. Also, unlike a number of articles, this article gives some consideration to the context in which a strike is occurring, noting that a strike would be far more difficult to justify at a time of national emergency such as during a pandemic
MacDougall^22^	2013	This article explores a key assumption in relation to the justification of strike action, that healthcare workers have a special relationship with society. The author examines common arguments that ground physicians’ special relationship with society and argues that such positions are untenable	Examining practice-based, utilitarian and social contract accounts of the relationship that healthcare workers have with society, this article argues that in grounding any ‘special obligations’, these positions are ‘either infeasible as views of medical morality…or are best understood as binding moral agents only when those agents have voluntarily submitted to the clear codes or traditions of self policing associations’		
Mawere^23^	2010	This article argues against a strike drawing on a range of ethical principles. Its most important contribution (for our purposes) and where it stands in contrast with the other articles included here, is that it provides an African perspective on these issues and draws on African communalism to argue that a strike cannot be justified	In arguing that a strike is not permissible, the author argues that a strike is ‘not only morally unjustifiable but also unfair and unjust to other members of the community. This is so because in any society (where people have the common goals) each member has his duties and responsibilities which s/he should accomplish with all the cogency, dedication and efficiency for his good and the good of the society…The values of individuals and individual rights, for example, are normally overridden by the values and rights of the community as a whole’		
Muyskens^24^	1982	This article argues for strike action on the grounds that nurses not only have obligations to their individual patients, but a collective obligation to maintain a high standard of care. In balancing these obligations, they suggest we imagine a modified Rawlsian original position, where ‘members of the public cannot know when or what nursing care they may need (they are under a veil of ignorance) and nurses also do not know in what situation they will find themselves’	This article argues that strikes can be justified as nurses not only have obligations to their patients, but a broader obligation to society in maintaining a high standard of care. The author essentially sees the most important consideration in weighing up whether it is justified as ‘how one balances the collective responsibility to maintain and improve the quality of nursing care with an individual nurse’s responsibility to her/his own patients’		
Neiman^25^	2011	This article argues that traditional deontological and consequential perspectives focus too narrowly on the tension a strike creates between nurse and patients. The author argues that healthcare is also a community endeavour, not just a conflict between nurses and their individual patients. That is, the community and a range of parties also have a responsibility for healthcare delivery. ‘The community as a whole has an obligation to provide healthcare for its members’	Similar to Muyskens above, this article argues that seeing a strike as a conflict between an individual nurse and their patient is myopic. The author argues that to understand and justify strike action, nurses need to be seen among broader healthcare systems, which are influenced by multiple parties such as insurance and government, for example. The author suggests that the responsibility for strike action extends beyond individual nurses		
Robertson and Bion^26^	2012	This is a debate article in which Robertson argues for strike action to protect doctors’ pensions, mainly on the assumption that patient care can be maintained. Bion presents the case against such action, arguing that such action would not only impact patients but may impact the standing of doctors more generally in the eyes of the public		The discussion presented in this article focuses on the possible consequences on strike action. Robertson, for example, believes that potential risks to patients can be mitigated and strike action is, therefore, justified. Bion, however, is more sceptical and not only raises patient care as an issue but the impact that such action could have on the standing of the professions more generally. Interestingly, Bion also takes on the position regarding responsibility for a strike. Unlike other authors who have argued that governments and the general public also have responsibilities for a functioning healthcare system, Bion suggests that this does not absolve healthcare workers of their responsibilities and, if anything, a focus on the government diminishes the professions as leaders. Bion also seems to suggest that such action could also contribute to a broader erosion of ‘professionalism’ in healthcare workers	Two issues regarding the nature of strike action are implied in this article. First, the goals of the action relate to doctors’ pensions. Second, one author believes the impact of such action on patients can be minimised (by continuing to provide a minimum standard of care), this point is disputed by Bion
Rosner^27^	1993	This article argues against a strike from a position of Jewish law, concluding that ‘a cardinal principle of Judaism is that life is of infinite value and clinicians cannot be justified in walking away from their posts’	This article argues that a strike cannot be justified because under Judaism, ‘a life is of infinite value and clinicians cannot be justified in walking away from their posts’. The argument advanced here, while grounded in Jewish law shares a number of parallels with more secular arguments above that healthcare workers have a ‘special obligation’ to society		
Selemogo^28^	2014	Drawing on just war theory, this article provides a framework against which strike action can be evaluated. The author argues that if action is justified it should meet each of the criteria laid out in this framework		Unlike other articles, instead of directly discussing the potential consequences of strike action, the author argues that instead it should be proportional. While proportionality is not discussed in much depth, it could be that the author is suggesting that a strike should be a proportional response to the problem at hand, it could also mean that a strike does not inflict unnecessary harm on patients	This framework goes on to outline a range of further considerations. This includes that a strike occurs for the right reasons; for the author, this generally means that a strike should seek to ‘confront a real and certain danger to the health of the population’. The author also argues that a strike should be a last resort, a minimum standard of care should be provided to patients throughout the strike, a strike should have a reasonable chance of being successful, that permission to strike has been granted from a central body (i.e. a union or professional body) and that a formal declaration is made, which the author appears to suggest could be used as a means to rally public support for the strike in question
Tabak & Wagner^29^	1997	This wide-ranging article discusses a number of elements of strike action. It discusses strikes as a ‘right or freedom’ on how the public view strikes and the legality of strike action. This article’s most interesting contribution for our purposes is that it focuses on the impact that strike action may have on individual nurses		This article notes that in past strike action, the public has found a scapegoat in nurses. The authors instead suggest that the government ought to take responsibility for why a strike is needed in the first place. The authors go on to discuss the potential risk of strike action for individuals, both nurses and the general public noting that, reaction to a strike is usually ‘based on ethical and moral claims, which play on nurses’ consciences’	This article goes on to discuss how a strike could be conducted to place patients at minimal risk. The authors argue that a minimum standard of care should be provided during strike action and that other healthcare workers are mobilised to assist. The authors also note that it is often the threat of a strike that is often enough to prompt action
Toynbee et al.^30^	2016	This article was written in the context of the UK junior doctors’ strikes. The authors argue against an absolute prohibition of strike action, noting that this would require the acceptance that doctors would have to work under any range of conditions at any time. The authors go on to outline the feature of strike action that would ensure it is justifiable, such as ensuring safeguards are in place to ensure patient well-being	The authors provide a practical explanation as to why an absolute prohibition on strike action is unsustainable and misguided, arguing that it would require an ‘acceptance that once a person becomes a doctor they are obliged to work under any conditions, at any time, with any number of patients’	This article argues that strikes under the right condition are not an unfortunate necessity, but necessary to address patient safety concerns. Again, and like many articles above, the authors use the issue of patient safety, but to argue for strike action. The authors also argue that the state also shares responsibility for such action	The authors argue that in this case, the demands attached to the strike were just, and that junior doctors in the United Kingdom at the time faced increasing pressures related to their workload. The authors go on to imply that a strike should be a last resort, and assume that a minimum standard of care will be left in place as consultants would be left to care for patients
Veatch & Bleich^31^	1975	This article outlines a debate between Veatch and Bleich. Veatch argues for strike action, turning to the principle of justice, noting that patient care may be sacrificed in the short term for long-term gains. Bleich, on the contrary, argues that immediate needs create immediate obligations and that strike action cannot be justified as healthcare workers possess a unique set of skills and, as a result, society can make unique claims on them	While this article largely focuses on the risks/consequences of strike action, it does touch upon why such action is justified or not. Veatch turns to the principle of justice to argue, like others above, that healthcare workers have a broader obligation to society, to future patients. Bleich. On the contrary, it suggests that as clinicians have a special set of skills, society can make special claims upon them. He does, however, acknowledge that society also has obligations, that they need to provide the systems and structures so that healthcare workers can discharge their duties	Veatch argues that a patient’s immediate interests could justifiably be compromised to serve a broader or future good. Veatch acknowledges that healthcare workers have entered into a ‘contract to render care’, however, contends that this is not without limits. Furthermore, Veatch also suggests that examining a strike as an individual issue oversimplifies the situation, arguing that, ‘[i]nsisting that the physician should do what he thinks will benefit those who are his particular patients at the present time is not only paternalistic and individualistic, it is also an oversimplified reduction of a complex set of social interactions. It defines the situation improperly’. Bleich, on the contrary, argues that ‘Immediate needs create immediate obligations. Anticipated needs do not generate immediate, compelling obligations’ and that as healthcare workers have a unique set of skills, society makes a unique claim on them	While neither author discusses the aims of strike action, it is assumed through this article that the aims of strike action are to improve patient care
Wolfe^32^	1979	This brief article provides individual reflections on strike action and offers an interesting perspective on who is responsible for such action. The author essentially argues that strikes can be justified if ‘the rights and health of patients and the public are preserved’ and that ‘health worker strikes, if his important caveat is respected, have in general not been shown to harm innocent people’		Perhaps the most interesting contribution of this article (for our purposes) is how the author frames the dilemmas of strike action. While supportive of such action if the rights of patients and the public can be maintained, Wolfe does not frame this as an issue that is for healthcare workers alone, noting that in many ways, healthcare workers are always on strike, with services withheld or inadequate for large groups of the population. Similar to Veatch above, the author appears to be appealing to justice, arguing that strike action may remedy existing inequalities and improve care for those who would otherwise not have it	

## Results

The papers included in this review represent a diversity of opinion about strike action, beginning to provide an overview of the complex ethical issues related to the justification of such action. For those that argued for a prohibition of healthcare strikes, positions ranged from arguing that a strike was never justified ‘regardless of the provocation’^17^ to calling for a ‘prima facie prohibition’^16^ on strike action. The difficulty in reaching this position was not taken lightly, for example, Counihan^12^ argued that despite being able to ‘identify with the striker, and indeed sympathise with him’ strike action could not be justified, drawing a military analogy, arguing that ‘[t]he sick and the wounded are regarded as outside the battlefield even in bitter and bloody conflicts’ and concluding that strike action was akin to ‘trying to cure a disease by administering poison’.^12^ On the contrary, however, a number of authors offered a passionate defence of strike action, reflecting on this costs of failing to act; Brecher^10^ argued that it is those against strike action ‘who bear the greatest responsibility, on their own grounds, for needless death and suffering’. The justification for these positions came down to the more fundamental issue of how authors conceptualised the relationship between healthcare professionals, their patients and society, the risks that they perceived came with strike action and the assumptions they made about how such action was conducted. These three themes will be the focus of the below synthesis.

### The relationship between healthcare workers, patients and society

One of the most fundamental issues that emerged from the literature related to how authors perceived the relationship between healthcare professionals, their patients and society. While many of the arguments that emerged here were closely related to the risks of strike action, namely to patients, a number of distinct arguments emerged related to strike action and whether it could be justified given how authors perceived what healthcare workers owe their patients and society.

Drawing on Jewish law, Rosner^27^ argued that ‘a cardinal principle of Judaism is that life is of infinite value and clinicians cannot be justified in walking away from their posts’. Similar arguments were echoed elsewhere with a number of authors asserting that because of their relationship to their patients, healthcare workers could not justify strike. These sentiments were perhaps best encapsulated by Glick^17^ who argued that ‘[h]ealth workers, and particularly physicians, are in a special class because they deal with human lives and because, upon joining the profession or accepting their job, they have voluntarily undertaken a commitment to those they serve’. This is put another way by Bleich, who in a debate article argued that

[p]hysicians possess skills which are not shared by other members of society. In accepting hospital appointments they agree to make their skills available to those whom they serve. Hence society has a unique claim upon their services and they, in turn, bear a unique responsibility to society.^31^

Similarly, Mawere^23^ draws on African communalism to argue that ‘where people share the same idea of personhood and communal life, physician strike is violation of the public trust—a complete failure to exhibit the prime duty and responsibility to other members of their community’. This position is somewhat distinct as the majority of those who argued that strike action could not be justified did so on the grounds that healthcare workers had a special relationship with their patients, not society as a whole.

In response to the above positions, a number of authors challenged the view that healthcare workers have some kind of special relationship with their patients and society. The first of these positions ranged from arguing that healthcare workers had no special relationship with their patients or society, to arguing that even if healthcare workers did have some kind of special relationship to their patients (for example), this could not be considered absolute. The second position argued that health and healthcare were collective endeavours, for which we all have a responsibility, that is, it is not just healthcare workers that have a duty to their patients, but that governments and society more generally have a responsibility to maintain a functioning healthcare system.

On the first of these points, Brecher^10^ responds by arguing that healthcare workers are not under any special moral obligation that would prevent them from striking, noting that ‘[u]nless we were all either to agree that human life is in all circumstances a completely overriding value…the striker whose omissions bring about someone’s death has no prima facie moral case to answer’. Loewy^21^ builds a similar case, arguing that healthcare is not the most important social good and the prohibition of strike action requires those making the argument to also show that healthcare is of paramount value. He notes that healthcare workers are equally as essential as those who work in garbage or waste disposal, and that ‘[u]ncollected garbage or unprocessed sewage are every bit as dangerous and have far more side-reaching health effects than do untreated pneumonia or appendicitis or coronary bypass surgeries that are not performed’. He also argues that while some of the tasks that healthcare workers provide are life-saving, many others are not. In a more recent article, MacDougall^22^ argues that the presumption above that health professions are morally special is often not defended and goes on to explore three prominent theoretical accounts that could ground such an assumption; practice-based, utilitarian and social contract accounts. He argues that such accounts are ‘either infeasible as views of medical morality…or are best understood as binding moral agents only when those agents have voluntarily submitted to the clear codes or traditions of self policing associations’. Others have pointed out the practical implications of placing health and healthcare above all other values, namely that it ‘requires an acceptance that once a person becomes a doctor they are obliged to work under any conditions, at any time, with any number of patients’.^30^

Turning to the second point, others have taken issue with the ‘hyper-individualistic’ way in which these issues have been framed, arguing that healthcare is a collective endeavour and that we all have an interest in ensuring that healthcare systems are well funded and healthcare workers well supported. For example, Neiman^25^ argues that nurses are often on the front line of what may be multiple systemic and structural failings for which others also bear responsibility, noting that arguments too often ‘focus narrowly on nurses and patients’. He argues that any decision to strike must be considered in context of their broader relationship with society, with this point made by considering this example:

There is not a linear chain of responsibility with a clear and identifiable cause on which to place moral blame for diminished quality of care. When insurance companies raise rates, fewer people are able to afford sufficient coverage. But whether this impacts the quality of care patients receive is dependent upon the ability and willingness of other parts of the healthcare community to make up for insurance companies’ decreased contribution. If, for example, hospitals increase their contribution by providing more charity care, or taxpayers increase their contribution by providing more funding for programs that serve the poor and uninsured, then insurance companies’ decision may have minimal impact on the overall quality of healthcare.

A similar argument is advanced by Chima^11^ who makes the point that it is not only healthcare workers that are responsible, but ‘the recognition by both employees and employers, especially elected officials that they are equally morally obligated to serve the interest of society’. Similarly, Muyskens^24^ argues that healthcare workers not only have responsibilities for their individual patients but a collective responsibility to maintain high standards of practice. He takes on Bleich’s point above, arguing that a strike is permissible; however, the most important consideration in weighing up whether it is justified relates to ‘how one balances the collective responsibility to maintain and improve the quality of nursing care with an individual nurse’s responsibility to her/his own patients’. Similarly, Veatch argues that

[i]nsisting that the physician should do what he thinks will benefit those who are his particular patients at the present time is not only paternalistic and individualistic, it is also an oversimplified reduction of a complex set of social interactions. It defines the situation improperly.^31^

The assumptions that were made about the relationship between healthcare workers, their patients and society often led to polarising opinions on strike action. Perhaps one of the biggest difficulties here in finding a way forward is that these arguments rest on some fairly unsettled beliefs regarding what healthcare workers owe their patients, society and vice versa. Turning to the empirical literature, there is actually very little known on how public and patients view strikes; however, what is available does not suggest that the general public or patients feel that such action should be prohibited.^33^ Furthermore, healthcare workers have never only had an absolute obligation to their patients, they of course have obligations to others; their employers and to society more generally, just to name a few. In saying this, at the other end of this spectrum, we should also be careful in dismissing the relationship that healthcare workers have with their patients and society, few would dispute that healthcare workers generally hold relatively trusted and privileged positions. Such arguments also often overlook a number of nuances. A doctor may have significantly different obligations for a patient in intensive care to one who requires non-urgent follow-up. Almost all strikes that have been documented in the literature detail at least some alternative arrangements made for patient care, even in strikes that lasted months. During the Israeli doctors’ strike in 1983, which last for over 4 months, emergency care remained in place and doctors who went on strike set up alternative clinics.^34^ Furthermore, the relationship between healthcare professionals, patients and society will change with time and context; for example, a pandemic may bring into focus further questions about this relationship. A recent example raises a series of questions for those opposed to strike action on the grounds that it violates healthcare workers relationship with their patients and society: should healthcare workers continue to work in Myanmar under a military government, during the COVID-19 pandemic, with inadequate personal protective equipment?^35^ To us, the most tenable position lies between these polarised positions, that is, while healthcare workers should prioritise patient care, this cannot be (and never has been) absolute; healthcare workers have a range of other obligations. Furthermore, health and healthcare are collective endeavours, for which we all have a responsibility, that is, it is not just healthcare workers that have a duty to their patients, but that governments and society more generally have a responsibility to maintain a functioning healthcare system and to provide healthcare workers with the means to carry out their jobs. While society can thus make claims on healthcare workers, they too can make claims on society. In saying all of this, however, this still says little about whether a strike could be justified; we also need to consider the consequences in taking such action and the related question of how strike action is conducted.

### The consequences of strike action

The issue that weighed most heavily throughout the literature included in this review related to the impact that strike action could have on patients. This issue weighed particularly heavily with those opposed to strike action. Dworkin,^15^ for example, argued that ‘[i]t surely must be impossible objectively to deny that grief, distress, physical harm and, almost certainly, unnecessary death must occur as the result of industrial action in the health service’. Glick^17^ offers similar reasoning, arguing that a strike cannot be justified as it will almost certainly harm patients. Maintaining this would be the case for any profession in which strike action may impact the health of others, he offers this analogy: ‘[i]f airline pilots threatened to parachute from their planes and leave their passengers without a pilot in mid-air that too is not acceptable. So too would be a strike of firemen or of employees in other vital services’. Some have taken a less dramatic stance. Counihan,^12^ for example, acknowledges that ‘[t]here are obviously gradations in the consequence of withdrawal of service by different groups in the service’. It is on the point that many have made a case for strike action, namely that the arguments against such action are overblown and simply do not reflect the realities of what a strike entails. A number of authors noted that strike action has never involved the walk-out of all staff and particularly those looking after patients who were acutely unwell.^21^ A number of other authors have asked us to think more broadly about issues of justice, not just about who is denied care because of a strike, but the consequences for those who do not have care more generally. Wolfe^32^ makes this point in the context of US healthcare, which is worth quoting in full:

…are not some doctors and some institutions always on strike? For example, is not the concerted, collective withholding of services from, say, fully insured persons unless they agree to pay extra fees, or from Medicare or from Medicaid, or from workers’ compensation recipients, actually a form of strike action? And, are not senior clinicians in teaching hospitals who often look after their private patients in one attractive part of their hospital or in their private offices, while their junior staff, interns, and residents look after the poor and the needy and the emergent cases in the traditionally shoddy outpatient clinics and emergency rooms-also exercising concerted, collective action in withholding their services from a broad segment of the patient population? These are difficult and value-laden questions, but they need to be asked. And, on the other hand, there are unjust laws and unjust decisions by federal, state, and municipal governments that may lead to injustices for those who need services.

Taking into account the consequences of failing to act and in acknowledging the potential consequences of strike action, a number of authors saw strike action as something that needed to be balanced against what it was trying to achieve. Selemogo,^28^ for example, framed these issues as one of proportionality, that is, strike action should be proportional to what it is hoping to achieve. Similarly, and on this point, a number of authors introduced a temporal element to the harms and risks of strike action, that is, can strike action be justified to avert harm to future patients. Veatch and Bleich,^31^ for example, argue that ‘[s]ometimes (but not always) the long-term interest of other patients or the physicianless must justify short-term compromises…’. Others have argued that compromises in patient care for future benefits are not uncommon in other areas of healthcare:

At times, advocating for ‘best care’ for future patients may mean compromising on ‘best care’ for current patients. There are already precedents for this. For example, renovating old facilities or replacing outdated equipment may improve the ability to care for patients in the future, but may temporarily reduce capacity to care for patients during the renovation or delay care during the transition from old to new equipment.^20^

To a much less extent, the other potential consequences of strike action, beyond that of impact on patients, were touched upon by a number of authors. Robertson and Bion,^26^ for example, argue (in the context of the United Kingdom) that industrial action is likely to diminish the authority of doctors and ‘enhance political arguments for creating a devolved and fragmented healthcare system in which collaboration is replaced by competition, and commitment by contracts’. While Fiester^16^ raises concerns regarding the ‘public’s respect for the medical profession’, Jackson^18^ suggests that ‘[i]f done for the right reasons and if conducted so that affected patients see their physicians seeking to preserve their identity as healers, then strikes potentially could strengthen physician–patient relationships at both the individual and collective level’. Dworkin,^15^ however, has other concerns, taking these points further, citing concerns that a strike could influence others to engage in similar acts, noting that a ‘general habit of obedience can drift into general habits of disobedience, which in turn are likely to upset dramatically the social and political balance of the country’. In the papers included in this review, few gave consideration to the risks and harms that strike action presents for healthcare workers themselves.^14^

When discussing the consequences of strike action, two quite polarised positions again appear to emerge, both to some degree, speaking to different parts of the problem. On one side, some have asserted that strike action ‘will almost certainly harm patients’,^17^ while likening such action to a pilot threatening to parachute from a plane while mid-air. Beyond risks to patients, some have argued broader consequences, such as diminishing trust in healthcare workers, or more dramatically, promoting more general disobedience. Such concerns are, of course, unfounded. The empirical literature suggests that strikes do not lead to an increase in patient mortality.^36^ While perhaps the airline pilot analogy could hold for staff caring for those critically unwell, like we discussed above, we are unaware of any healthcare strike that has simply resulted in all staff walking off the job and leaving those who are most in need of care. In saying this, the risks with strike action go far beyond that to individual patients; this was overlooked by a number of articles included in this review. Most articles included in this review came from the global North, in generally higher income countries and failed to consider the risks that strike action may have for healthcare workers, beyond damage to reputation or public trust. Looking only to the last few months in Myanmar, healthcare workers have taken significant risks in going on strike and in treating protesters, with some going into hiding and others being attacked and shot.^37^ Medical students in Ecuador were met with tear gas after demanding they be paid a salary for their work during the COVID-19 pandemic.^38^ Perhaps more problematically though, discussions about the consequences of strike action only get us so far. As can be seen from the above articles, the argument for the potential harms and risks related to strike action cuts both ways. Those against strike action have argued against it on the grounds of the potential risks it presents to patients; however, those who are for strike action argue that these risks and harms can be proportional (and can be mitigated). Furthermore, those who argue for a strike generally highlight broader harms and risks related to the healthcare system more generally and for future patients. Put another way, arguments can be made for or against a strike on the grounds of patient harm. It could be argued that a strike is not justified because of the harms it could do the patients; however, an argument could be made that current arrangements that harm patients justify such action or that such action in the longer term would lead to less harm to patients. These debates have occurred outside of the literature as well. In Australia, for example, where nurses undertook strike action demanding better conditions and patient safety, the Australian government ‘repeatedly used “patient safety” to name, blame and shame the nurses for their action and to falsely attribute the “everyday” deficits and failings of the health care system to the industrial action being taken’.^19^ It is, of course, plausible that a strike could harm patients, it is also completely plausible that a strike may have few adverse impacts for patients. It could be argued that on balance, a strike would be better in the long run and any negative consequences would lead to long-term benefits. To make a case either way, we need to look at the nature of the strike itself, that is, the consequences of strike action will largely depend on how it is conducted.

### Conducting strike action

While there were fewer papers that examined issues related to the conduct of strike action, we can begin to identify some of the key characteristics raised in relation to the justification for such action.

One issue that was more frequently raised than others were the reasons for pursuing strike action, or in other words, the demands such action makes. For Daniels,^13^ this was a particularly important consideration, arguing that ‘[f]rom a moral point of view it is far more important to worry from the start about the justice of the goals doctors seek than it is to worry about their “right” to bargain collectively for their goals’. He goes on to note that it would be difficult to justify a strike unless a ‘significant part of their goals demands directly related to improved patient care’. On this point, what should the goals of strike action be? A number of authors have assumed that a strike is generally undertaken to improve patient care, for example, Li et al.;^20^ while others have spoken about demands in the context of a specific episode of strike action.^30^ Others have made explicit the reasons as to why a strike may be justified. Selemogo^28^ argues that a strike should only be carried out ‘to confront a real and certain danger to the health of the population’. Veatch and Bleich^31^ also believe that improving patient care should be a central consideration; however, turning to the principle of justice means that a strike could be justified more broadly to consider the healthcare system more generally and the needs of future patients. One problem that was often overlooked was the fact that motives for strike action vary and they are often mixed. Loewy^21^ suggests that both motives to improve patient care and out of self-interest are both justifiable, arguing,

in fairness, workers are entitled to the fruits of their labor, fruits that should amply reflect the value of their work and their share of the profits. Physicians and nurses often strike to create better conditions for their patients as well as better conditions for themselves: neither reason is ethically to be decried.

A further issue that was discussed related to the safeguards put in place during a strike. That is, the alternative arrangements for patients and services that remain in place during the strike. Even those most sympathetic towards strike action, almost all agreed that emergency care should remain in place and where possible, for those in need of less acute care, alternatives should be provided. For example, Chima^11^ argued healthcare workers ‘must endeavour to provide a certain level of minimum service’. Recognising the dynamic nature of strike action, Li et al.^20^ argue that ‘[t]o minimize patient harm, striking physicians often exercise substantial discretion in the intensity and duration of withdrawal of patient services’. Perhaps unsurprisingly, those who feel strike action should be prohibited were sceptical that any safeguards could be put in place. Counihan,^12^ for example, argues that ‘[w]e sometimes like to blur the picture and perhaps salve our consciences by providing services for emergencies only. This is a very nebulous concept’.

Two further issues also emerged. First, whether a strike is a last resort, that is, have all other avenues been exhausted before reverting to a strike. Second, whether a strike has a reasonable chance of having its demands met. Both of these issues are as much pragmatic as they are ethical; however, they deserve consideration as both could influence the trajectory of strike action, and its likelihood of having its demands met and therefore the risk it presents to patients. In relation to being a last resort, Daniels^13^ argues that it would be ‘hard to justify such strikes if there were any other way of achieving the goal that imposed less burden and risk on the patient population’. Selemogo^28^ calls for all ‘less disruptive’ alternatives to be exhausted while Li et al.^20^ calls for a strike only after there is no alternative, after ‘repeated good-faith efforts at negotiation’. Finally on this point, Tabak and Wagner^29^ argue that it is often not a strike itself that is impactful, but the threat of strike action, with the threat of a strike alone ‘generating strong differences of opinion, unrest within the health system, wasted work days spent on discussion and planning, the recruiting of paramedical staff, mutual accusations, and the harsh exposure of flaws in the system by the media’. While the second issue received less attention, a number of authors also argued that the likelihood that the demands of a strike would be met should factor into decision-making. For example, Selemogo^28^ argues that a strike should have at least some chances of success to be justified. Beyond these two points above, there have been a small number of issues noted, but have received less attention. A number of authors have raised the issue of public support, for example, Daniels,^13^ recognising that the support of the public is also far more likely to lead to a strike’s demands being met and for the strike to end quickly. Selemogo^28^ also calls for two further criteria to be met before a strike is justified, namely that a strike is sanctioned by some kind of official group, such as a union or association, as a further safeguard to healthcare workers and that prior to a strike being undertaken a formal declaration is made, which for Selemogo^28^ appears to be a further means to ensure public support for the strike.

Discussions related to the conduct of strike action appear to have the most promise in advancing our understanding about the justification for such action. As we have noted above, we feel arguments that dismiss strike action because of healthcare workers ‘special’ relationship with their patients (or society) are unconvincing; we also believe that discussions about the risks of strike action need to be placed in context. Most simply, we cannot begin to approximate the risks of strike action without having some idea of how a strike is conducted. As can be seen from the many examples in the introduction of this article, strike action in healthcare varies substantially; in most cases, care is maintained for those most unwell and alternative arrangements are often made for other services. In first defining how such action is conducted, we can better approximate its impact, given the context in which it is occurring. In saying this, there are still a number of shortcomings that appear to emerge here. For example, most authors appear to make assumptions about the demands attached to strike action and few discussed the dynamic and often mixed motives that come with such action. We also feel that some of the papers here are overly restrictive, dismissing strike action on unreasonable grounds. Selemogo,^28^ for example, argues that a strike should not be undertaken for ‘self-enrichment’. While a strike may be more difficult to justify on these grounds for those who are paid well and work under relatively good conditions, could we also argue this is the case for doctors and nurses in Zimbabwe, who on average earn the equivalent of US$30 and US$115 a month, respectively?^39^

## Discussion

Strikes remain a contentious issue that have, over decades, drawn passionate and polarising debate. In the above review, we set out to answer three questions, namely to outline the reasons given regarding the justifiability of strike action in the literature, the relative strengths and shortcomings of this literature and the future directions that this provides. Those who have generally argued against such action, cite the harm that strike action and, in particular, its impact on patients. Many also argue that healthcare workers because of their skills and position in society, have a special obligation to their patients and society more generally. Those who see the strike action as not only permissible but also, in some cases, necessary have advanced several points in response, arguing that healthcare workers do not have any special obligation to their patients or society, more than any other worker does and even if this is true, this obligation is not limitless. Those who argue against a strike often frame the issue as one between a healthcare worker and patient, and that ultimately healthcare workers are responsible for such action, those who are sympathetic to such action generally frame these issues much more broadly, arguing that we all have a responsibility in maintaining a functioning healthcare system, and that it is healthcare workers that are on the end of multiple structural failings.

Overwhelmingly, when talking about the potential risks of strike action, authors have focused on patient welfare and the impact that a strike could have. As noted above, this is most frequently cited as the reasons why a number of authors oppose such action. Others paint a more complex picture, not only arguing that the view that a strike is undoubtedly going to harm patients is overblown, introducing ideas of proportionality and arguing that any risks associated with a strike need to be balanced against failing to act. A number of other risks have been identified such as the broader impact that such action could have on the healthcare profession as a whole, for example, damaging public trust.

One issue that becomes apparent is that arguments based on risk alone do little to advance the question of whether a strike can be justified. The literature here is often disconnected from the empirical literature related to the impact of strike action and, furthermore, overlooks the fact that the risks of strike action can vary depending on the context in which it is carried out and the nature of the action itself. These issues have received less attention, but remain important. A number of authors note that factors such as the length of a strike, the staff who go on strike, the demands of a strike are all as important in considering its justification.

While we have provided some critical reflection throughout, these issues are worth summarising and expanding upon here. Many of the articles included in this review, dismissed strike action on the grounds of the relationship healthcare workers had with their patients and society. Such positions, however, are unconvincing. While healthcare workers should prioritise patient care, this cannot be (and never has been) absolute; healthcare workers have a range of other obligations. In addition, health and healthcare are collective endeavours, for which we all have a responsibility, that is, it is not just healthcare workers that have a duty to their patients, but that governments and society more generally have a responsibility to maintain a functioning healthcare system and to provide healthcare workers with the means to carry out their jobs. Most articles also discussed the consequences of strike action. Majority of these discussions included assumptions about what strike action was and how it was conducted. While we feel careful consideration should be given to the consequences of strike action (for patients and society, more broadly), the most productive way to start this conversation appears to be with how a strike could be conducted; its demands, who goes on strike, for how long and how care for those in most need could be maintained and the context in which it is occurring. This puts us in a far better position to begin to discuss the potential consequences of such action.

### Limitations

On this point about limitations, we should also acknowledge the limitations of this review. Above, we have presented a summary of the major arguments for and against strike action, which we have attempted to do so in a transparent and systematic way; however, we cannot be certain that the arguments we presented above are exhaustive or represent every distinct contribution to the literature. While far from agnostic to strike action, and while we believe some arguments have more merit than others, our conclusions and critique remain relatively broad, there is potential here for greater critique of the literature in a more focused review; this moves us to our final point, what can be learnt from this literature for future discussions, and theoretical and empirical research.

### Future Directions and Conclusions

Given the frequency and high stakes nature of strike action, it is perhaps surprising there has not been more discussion on these issues. Needless to say, there is scope to advance this literature in a number of ways. Many of the issues related to whether a strike is permissible relate to fundamental assumptions in what it is that healthcare workers owe to their patients and society. While there has been substantial discussion on this topic more generally, we know relatively little in regards to how healthcare workers and, in particular, patients and the public perceive healthcare strikes. Arguments could be made from this position that healthcare workers have both obligations to their patients and society more broadly, particularly in maintaining a functioning healthcare system, for example. On the contrary, it could be argued that a healthcare worker’s overriding obligation is to their patient. Greater work could be done to explore these assumptions along with their implications related to strike action. There also appears to be greater scope to explore how structural and systemic issues impact strike action. While a number of authors have argued that strike action is not solely an individual responsibility and instead usually due to multiple structural failings, there is scope to probe this point in theoretical and empirical work and how historical, structural, social and systemic factors influence strike action, for example, the study by Kowalchuk.^40^ Further attention should also be given to how a strike is conducted, more could be said about the context in which strikes occur, their demands, contingencies put in place during strike action and how these actions are framed. In advancing their arguments, a number of papers examined here appear to have made assumptions about the nature of strike action, for example, doctors being well-paid. While true in most of the global North, this cannot be said everywhere in the world. It may be that doctors in certain parts of the world are less justified in striking for increases in pay than others, in lower income countries, for example. It may be that striking is not justified in authoritarian countries because of the risks it carries. Furthermore, little has been said about the dynamic nature of strike action, particularly for those which are protracted; risks, demands and the nature of the strike can often evolve, shifting the calculus as to whether such action is justified. Closely related to this point, there is a need to tie this literature in with the existing empirical evidence. Over a number of decades, empirical evidence about the impact of strike action has grown; broadly, this literature examines the impact of strikes on patient outcomes and healthcare delivery. While it is beyond the scope of this article to discuss this literature in any detail, it should be said that this literature does not paint a clear picture about the impact of strike action and, if anything, there are a number of studies that have shown that if contingencies are put in place, patient outcomes are minimally impacted as are the delivery of services.^41,42^

Over the last several decades, strike action in healthcare has been common, even over the last 18 months, during the COVID-19 pandemic, the world has arguably witnessed an uptick in strikes and unrest among healthcare workers.^43^ These issues are unlikely to dissipate, with the ongoing impact of the pandemic, along with decades of neglect combining to present unprecedented challenges for healthcare workers. We hope that the above review begins to not only shed light on some of the more controversial issues related to such action, but also to provide some direction in moving conversations forward on these issues. Strike action will unfortunately remain a feature of many health workplaces into the foreseeable future; questions about how such action can be undertaken while minimising the risk to patients and others remain as pressing as ever.
